# Clinical implication of thoracic skeletal muscle volume as a predictor of ventilation-weaning failure in brain-injured patients: A retrospective observational study

**DOI:** 10.1097/MD.0000000000035847

**Published:** 2023-10-27

**Authors:** Jimi Oh, Hyun Lim, Chang Won Jeong, Min Su Kim, Jinseok Lee, Wu Seong Kang, Ui Ri An, Joo Un Park, Youngick Ahn, Youe Ree Kim, Chul Park

**Affiliations:** a Department of Anesthesiology and Pain Medicine, Ulsan University Hospital, University of Ulsan College of Medicine, Ulsan, Republic of Korea; b Division of Pulmonary Medicine, Department of Internal Medicine, Wonkwang University School of Medicine, Iksan-si, South Korea; c Smart Health IT Center, Wonkwang University Hospital, Iksan-si, South Korea; d Department of Rehabilitation Medicine, Soonchunhyang University, College of Medicine, Cheonan-si, South Korea; e Department of Biomedical Engineering, Kyung Hee University, Yongin-si, South Korea; f Department of Trauma Surgery, Cheju Halla General Hospital, Jeju-si, South Korea; g Department of Radiology, Wonkwang University School of Medicine, Iksan-si, South Korea.

**Keywords:** brain injury, computed tomography, mechanical ventilation, respiratory volume depletion, sarcopenia, tracheostomy

## Abstract

Sarcopenia, a generalized loss of skeletal muscle mass that is primarily evident in the respiratory musculature, is associated with adverse outcomes in critically ill patients. However, the relationship between sarcopenia and ventilation-weaning outcomes has not yet been fully studied in patients with brain injuries. In this study, we examined the effect of reduced respiratory muscle mass on ventilation weaning in patients with brain injury. This observational study retrospectively reviewed the medical records of 73 patients with brain injury between January 2017 and December 2019. Thoracic skeletal muscle volumes were measured from thoracic CT images using the institute’s three-dimensional modeling software program of our institute. The thoracic skeletal muscle volumes index (TSMVI) was normalized by dividing muscle volume by the square of patient height. Sarcopenia was defined as a TSMVI of less than the 50th sex-specific percentile. Among 73 patients with brain injury, 12 (16.5%) failed to wean from mechanical ventilation. The patients in the weaning-failure group had significantly higher sequential organ failure assessment scores [7.8 ± 2.7 vs 6.1 ± 2.2, *P* = .022] and lower thoracic skeletal muscle volume indexes [652.5 ± 252.4 vs 1000.4 ± 347.3, *P* = .002] compared with those in the weaning-success group. In multivariate analysis, sarcopenia was significantly associated with an increased risk of weaning failure (odds ratio 12.72, 95% confidence interval 2.87–70.48, *P* = .001). Our study showed a significant association between the TSMVI and ventilation weaning outcomes in patients with brain injury.

## 1. Introduction

Patients with brain injury require mechanical ventilator (MV) support to prevent aspiration and correct hypercapnia and hypoxemia, which can lead to secondary brain damage. Although ventilatory support may be lifesaving in patients with brain injury, it may also be associated with significant complications such as barotrauma, ventilator-associated pneumonia, ventilator-induced lung injury, and airway injury.^[1,2]^ Thus, it would be safe to discontinue MV as soon as possible. On the other hand, premature discontinuation can lead to deleterious effects; weaning failure or subsequent reintubation has been independently associated with consistently adverse clinical outcomes in many studies.^[3–6]^ Unfortunately, the commonly used traditional “weaning parameters” for extubation perform poorly in brain-injured patients.^[7]^ In the case of high-level, general hospital, university, or tertiary hospital that prioritize acute phase treatment, different ventilator weaning process are performed in each intensive care unit (ICU), which varies by country and continent. For these reasons, there have been few reports regarding factors associated with weaning success in patients with brain injury, some studies have included patients in stabilization or rehabilitation centers rather than the ICU. Therefore, the approach to the weaning procedure in brain-injured patients is still challenging for clinicians.

Sarcopenia is the generalized loss of skeletal muscle mass and function. Although the development of sarcopenia is mainly related to aging, it has recently attracted attention in the management of patients with brain injuries such as stroke. Brain injury-associated sarcopenia and modifications of respiratory function may explain an increased risk of ventilation-weaning failure in brain-injured patients.^[8]^ Sarcopenia may adversely affect ventilation weaning outcomes, especially in patients with brain injury, as sarcopenia may cause respiratory muscle dysfunction.^[9,10]^ However, sarcopenia is underemphasized or unrecognized as a potential predictor of ventilation-weaning failure in brain-injured patients. Therefore, we sought to determine whether sarcopenia adversely affects the outcomes of patients with brain injury admitted to the ICU. We hypothesized that sarcopenia might predict weaning failure in patients with brain injury.

## 2. Methods

### 2.1. Study population

**Figure 1.**
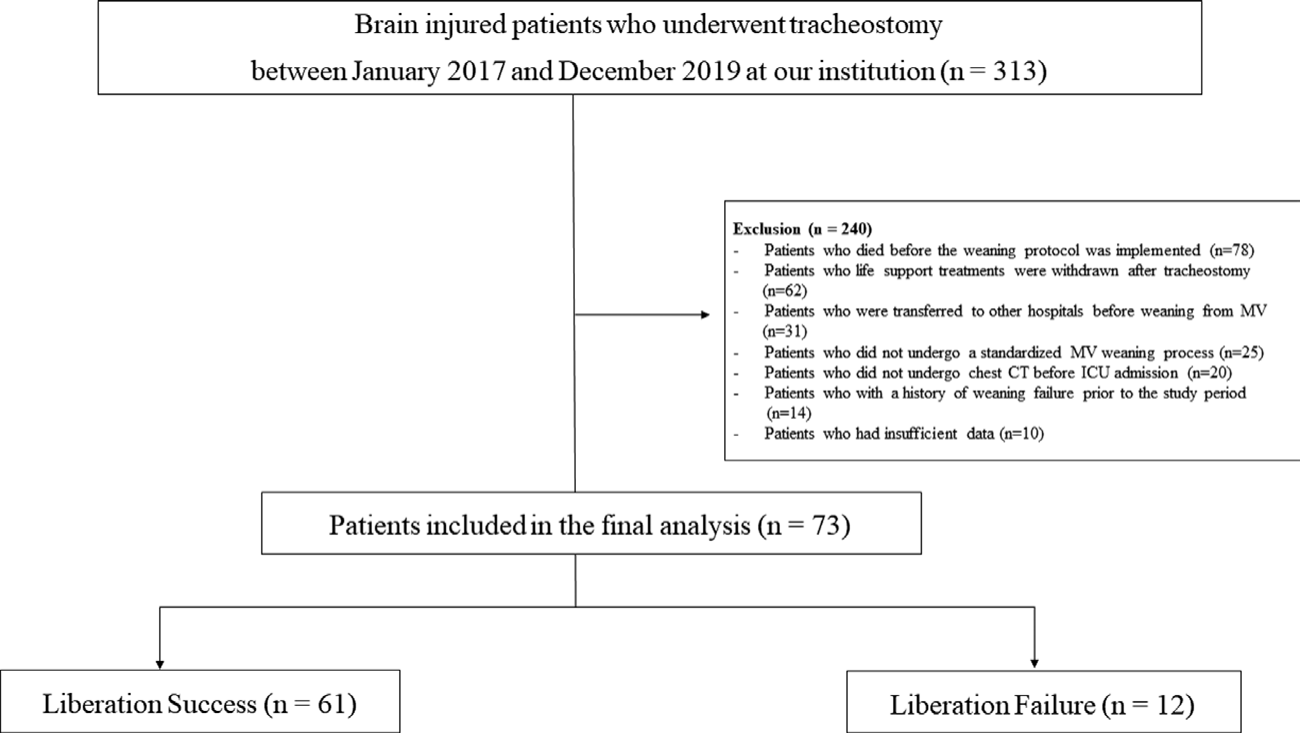
Study flowchart.

This single-center, retrospective study was approved by the Institutional Review Board of our institution (WKUH IRB 2020-09-040), and the requirement for informed consent was waived due to the observational nature of the study. We identified the medical records of patients with acquired brain injury (aged ≥ 19 years) admitted to the medical or trauma ICU at Wonkwang University Hospital (a 798-bed, university-affiliated, tertiary referral hospital in Iksan, South Korea) between January 2017 and December 2019.^[11]^ Among patients with brain injury, only those with a performance status ≥ 3 according to the Eastern Cooperative Oncology Group, a Karnofsky scale score of < 50 points, and a Zubrod Scale score of 3 or 4 were included in our study. The performance status of these patients corresponded to the extent to which they could perform certain daily living activities with assistance from others. Patients with post-cardiopulmonary resuscitation hypoxic brain injury, traumatic brain injury, cerebral hemorrhage, cerebral infarction, status epilepticus, meningitis, Parkinson disease, and Alzheimer dementia were included in this study. Tracheostomy was performed in patients who required prolonged mechanical ventilation (PMV) support for >7 days. The exclusion criteria were as follows: death before the weaning protocol was implemented, withdrawal of life support treatments after tracheostomy, nonstandard MV weaning process, ventilatory weaning failure prior to the study period, and missing data. Finally, 73 patients were enrolled in this study (Fig. 1). All data were reviewed using an electronic medical record system, including baseline demographic characteristics, laboratory data upon ICU admission, comorbidities, clinical outcomes, and mortality.

### 2.2. Standardized MV weaning process

The same ICU physician evaluated the readiness to liberate patients from MV following the institution’s standardized weaning protocol, which included 3 components. First, the indications for tracheostomy, brain injury, mental state, use of sedative drugs, vital signs, avoidance of positive fluid balance, and vasopressor requirements were evaluated. Second, a daily spontaneous breathing trial (SBT) was conducted by either a respiratory physician or the ICU staff. During the SBT, adequate gas exchange was monitored (PaCO_2_ < 45 mm Hg, PaO_2_ > 75 mm Hg) at FiO_2_ < 40% with a positive end-expiratory pressure below 8 cm H_2_O. Hemodynamic stability (≤0.05 μg/kg/min norepinephrine or ≤5 μg/kg/min dopamine and dobutamine) is permitted up to 24 hours before the weaning trial. Third, the attending physicians evaluated the weaning parameters, including the rapid shallow breathing index (RSBI), P0.1, and maximal inspiratory pressure. The RSBI was used to predict the ventilatory performance at 105. Patients with an RSBI < 105 are more likely to wean from the MV or succeed in spontaneous breathing.

### 2.3. Computerized measurements of thoracic skeletal muscles volume and outcomes

**Figure 2.**
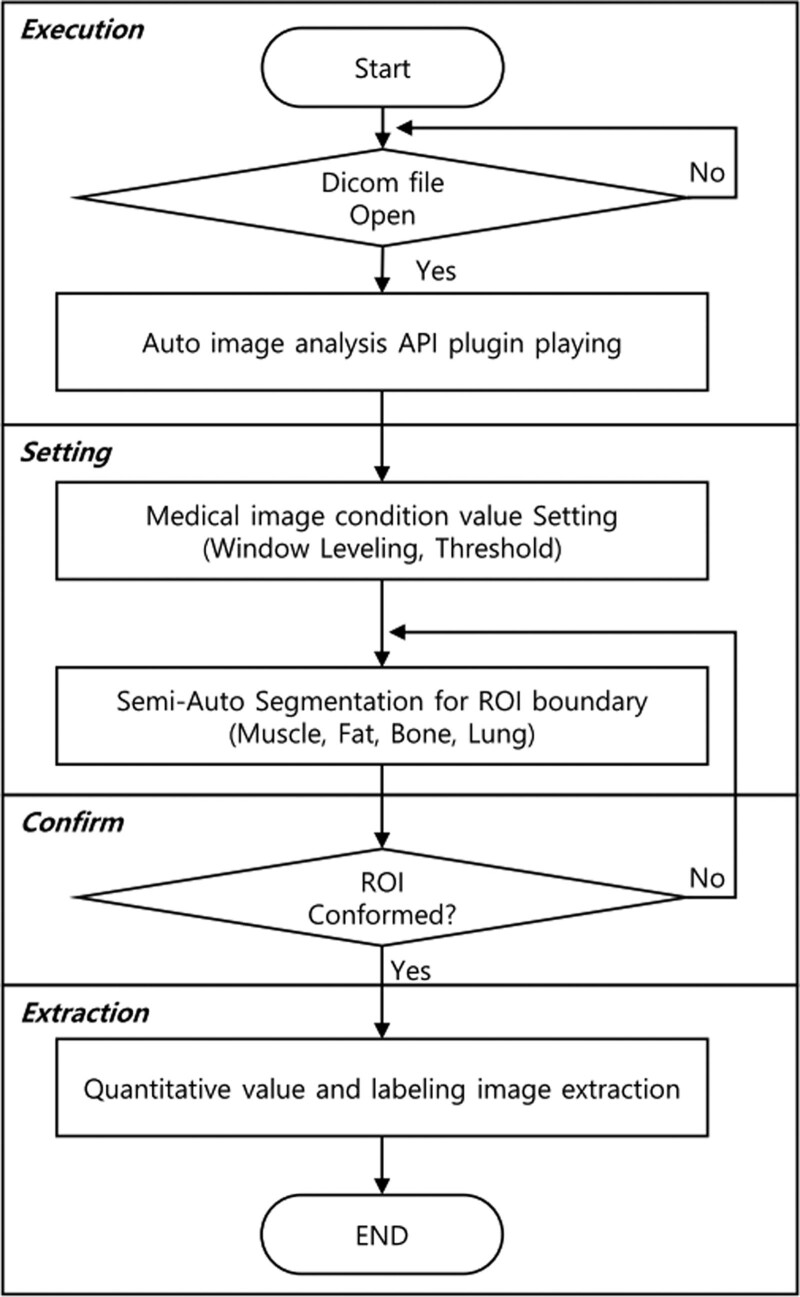
Process of image analysis software. API = application process interface, ROI = region of interest.

**Figure 3.**
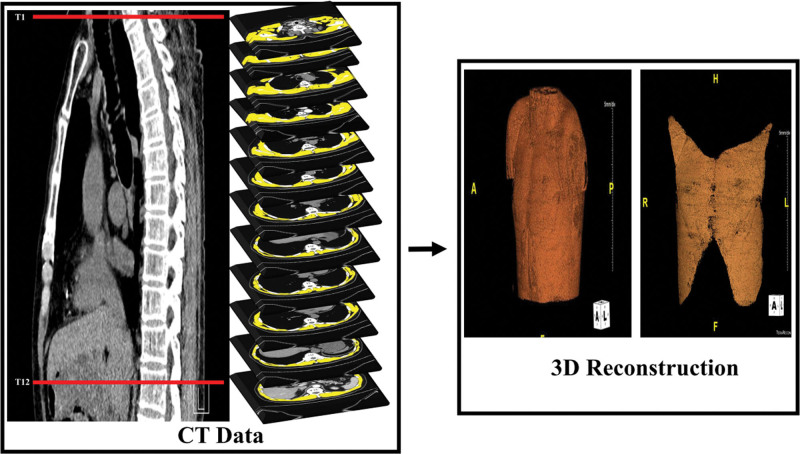
Thoracic skeletal muscles and 3D reconstruction.

Routine chest CT was performed for all patients in the emergency room before ICU admission. Sarcopenia was evaluated by measuring the thoracic skeletal muscle at the thoracic vertebral level using chest CT. All images were analyzed using WonkwangJ developed based on ImageJ Open Source (RRID: SCR_003070).^[12]^ A specific area range was designated to analyze anatomical elements. The results were improved using a semiautomated method to obtain the result using one or more images. The Hounsfield unit value of the CT image was set to an optimized threshold for the muscle area. Images of the regions of interest (ROIs) of the muscles were extracted and digitized. The image was processed in 4 stages: execution, setting, confirmation, and extraction (Fig. 2). During the execution stage, the medical image (DICOM file) to be analyzed was read using open-source ImageJ software. The window, leveling, and threshold values were determined to generalize the CT images during the setting stage. During the confirmation stage, the ROI for each area was confirmed using the generated semiautomatic method described above. The quantitative analysis value of each significant composition content volume in the ROI was quantified, stored, and provided as a comma-separated value file during the extraction phase. As the spine level scanned for chest CT in this study differed for each patient, each thoracic skeletal muscle volume was measured using images from the thoracic vertebrae 1–12. Using 1.0 mm slice thickness data from a multi-slice spiral CT scanner, the volume of the thoracic skeletal muscle (mm^3^) was measured in numerous trans-axial pictures at the level of the thoracic spine. Prior segmentation is necessary for these metrics. Manual outlining of the muscles is the simplest segmentation technique. Then, a normalized index value for measured muscle volume was calculated using squared body height, which resulted in the thoracic skeletal muscle volume index (TSMVI) (Fig. 3). All CT images were analyzed by a radiologist specializing in musculoskeletal systems who was blinded to the clinical data. As there are no diagnostic criteria or cutoff value for the TSMVI to define sarcopenia in patients with brain injury, we defined sarcopenia as TSMVI less than the 50th sex-specific percentile. The primary outcome was ventilator-weaning failure, defined as the need for ventilator support within 48 hours after SBT. The secondary outcomes were the length of ICU stay, length of hospital stay, and in-hospital mortality.

### 2.4. Statistical analyses

Continuous variables were presented as median (interquartile range) or mean ± standard deviation, and dichotomous variables were presented as absolute numbers (proportion). Comparisons of dichotomous variables among groups with or without ventilator weaning success were analyzed using the chi-squared test or Fisher exact test; continuous variables were compared using the Student *t* test or Mann–Whitney *U* test. Regression analyses were used to identify the variables associated with weaning from MV. Statistically significant variables in the univariate logistic regression analysis were subjected to a multivariate analysis. Additionally, the receiver operating characteristic curve was examined to assess the ability of sarcopenia to predict ventilator weaning outcomes, and a cutoff point for predicting ventilator-weaning failure using the Youden index method was identified. All tests were two-tailed, and *P* values < 0.05 were considered statistically significant. All analyses were performed using SPSS (version 24.0; RRID: SCR_016479).

## 3. Results

### 3.1. Characteristics of ventilator-weaning success and failure groups

During the study period, 313 patients in ICUs were screened for eligibility, of whom 73 were enrolled in the study. The baseline characteristics and clinical outcomes of the patients requiring PMV according to weaning success or failure are shown in Table 1. The mean age was 70.3 ± 15.1 years, and 69.9% were male. The distribution of patients according to brain injury type is as follows. Twenty-four patients (21.9%) were post-cardiopulmonary resuscitation hypoxic brain injury, 15 patients were traumatic brain injury (20.5%), 9 patients (12.3%) were cerebral hemorrhage, 8 patients (11.0%) were cerebral infarction, 2 patients (2.7%) were status epilepticus, 2 patients (2.7%) were meningitis, were 5 patients (6.8%) Parkinson disease, and 8 patients (11.0%) were Alzheimer dementia were included in this study. Sixty-one patients (83.5%) were successfully liberated, whereas weaning failed in 12 (16.5%). The patients in the weaning-failure group had a significantly higher SOFA (sequential organ failure assessment) score [7.8 ± 2.7 vs 6.1 ± 2.2, *P* = .022] and lower TSMVI [652.5 ± 252.4 vs 1000.4 ± 347.3, *P* = .002] compared with those in the weaning-success group. Sarcopenia in the weaning failure group was significantly higher than that in the weaning success group (58.3% and 13.1%, respectively; *P* < .002). However, there were no significant differences in the other demographic and clinical data between the groups. For clinical outcomes, the length of hospital stay was significantly longer in patients who failed weaning attempts than in those who were successfully weaned [50.0 ± 27.8 days vs 33.2 ± 13.2 days, *P* = .029]. However, the length of ICU stays [27.5 ± 20.9 days vs 25.2 ± 24.9 days, *P* = .729] and in-hospital mortality rates [8.3% (1/12) vs 19.7% (12/61), *P* = .599] did not differ between the 2 groups.

**Table 1:** Demographic characteristics upon ICU admission and clinical outcomes of study patients.

Parameters	Total (N = 73)	Liberation success (N = 61)	Liberation failure (N = 12)	*P* value
Age, years	70.3 ± 15.1	69.7 ± 15.7	73.5 ± 12.1	.433
Female	22 (30.1)	18 (29.5)	4 (33.3)	.928
BMI, kg/m^3^	21.6 ± 4.2	22.0 ± 4.3	19.6 ± 3.2	.069
*Comorbidities*				
Diabetes	25 (34.2)	23 (37.7)	2 (16.7)	.248
Malignancy	8 (11.0)	6 (9.8)	2 (16.7)	.852
Respiratory	6 (8.2)	5 (8.2)	1 (8.3)	.682
Renal	9 (12.3)	8 (13.1)	1 (8.3)	.879
Cardiovascular	12 (16.4)	9 (14.8)	3 (25.0)	.401
Gastrointestinal	6 (8.2)	4 (6.6)	2 (16.7)	.312
*Severity at ICU admission*				
SAPS 3	69.7 ± 12.6	69.9 ± 12.7	68.6 ± 12.1	.062
SOFA	6.5 ± 3.1	6.7 ± 3.0	5.7 ± 3.5	.654
LIS	1.0 [0.5–1.8]	1.3 [0.7–2.0]	0.9 [0.0–1.6]	.168
*Cause of brain injury*				
Post-CPR hypoxic brain injury	6 (8.2)	5 (8.2)	1 (8.3)	.903
Cerebral hemorrhage	43 (58.9)	35 (57.4)	8 (66.7)	.514
Cerebral infarction	20 (27.4)	17 (27.9)	3 (25.0)	.631
Others (epilepsy, seizure, meningitis)	8 (11.0)	6 (9.8)	2 (16.7)	.368
Parkinson disease	12 (16.4)	10 (16.4)	2 (16.7)	.401
Dementia	13 (17.8)	12 (19.7)	1 (8.3)	.093
*Reasons for ICU admission*				
Cardiology	5 (6.8)	4 (6.6)	1 (8.3)	.942
Pulmonary	38 (52.1)	30 (49.2)	8 (66.7)	.428
Sepsis or septic shock	2 (2.47)	2 (3.3)	0 (0.0)	.887
Post-operation	5 (6.8)	5 (8.2)	0 (0.0)	.687
Neurology	7 (9.6)	6 (9.8)	1 (8.3)	.482
Trauma	16 (21.9)	14 (23.0)	2 (16.7)	.921
*Parameters at procedure day*				
PT (INR)	1.2 ± 0.1	1.2 ± 0.2	1.2 ± 0.1	.726
APTT, seconds	32.8 ± 9.0	32.3 ± 8.8	35.7 ± 10.2	.232
Platelet count, x 10^3^/μL	253.3 ± 116.1	257.6 ± 118.1	231.7 ± 107.5	.484
Time from intubation to tracheostomy, days	8.77 ± 7.96	8.46 ± 7.22	8.66 ± 7.65	.650
SOFA	6.4 ± 2.4	6.1 ± 2.2	7.8 ± 2.7	**.022**
LIS	1.35 ± 0.77	1.47 ± 0.75	1.39 ± 0.76	.526
Driving pressure	35.58 ± 15.85	39.71 ± 10.07	37.03 ± 14.10	.383
PFR	272.13 ± 112.71	299.88 ± 125.3	257.08 ± 118.26	.145
Respiratory muscle volume index, cm^3^/m^2^	943.2 ± 356.5	1000.4 ± 347.3	652.5 ± 252.4	**.002**
Sarcopenia	15 (20.5)	8 (13.1)	7 (58.3)	**.002**
*Clinical outcomes*				
ICU LOS, days	27.2 ± 21.4	25.2 ± 24.9	27.5 ± 20.9	.729
Hospital LOS, days	47.2 ± 26.7	33.2 ± 13.2	50.0 ± 27.8	**.003**
In-hospital mortality	13 (17.8)	12 (19.7)	1 (8.3)	.599

Continuous variables are presented as mean (standard deviation). Categorical variables are presented as number (%). Bold type indicates statistical significance.

APTT = active prothrombin time, BMI = body mass index, CPR = cardiopulmonary resuscitation, CT = computed tomography, ICU = intensive care unit, LIS = lung injury score, LOS = length of stay, NOAC = new oral anticoagulant, PFR = PaO_2_FiO_2_ ratio, PT = prothrombin time, SAPS = simplified acute physiologic score, SOFA = sequential organ failure assessment.

### 3.2. Predictors of weaning from MV

**Figure 4.**
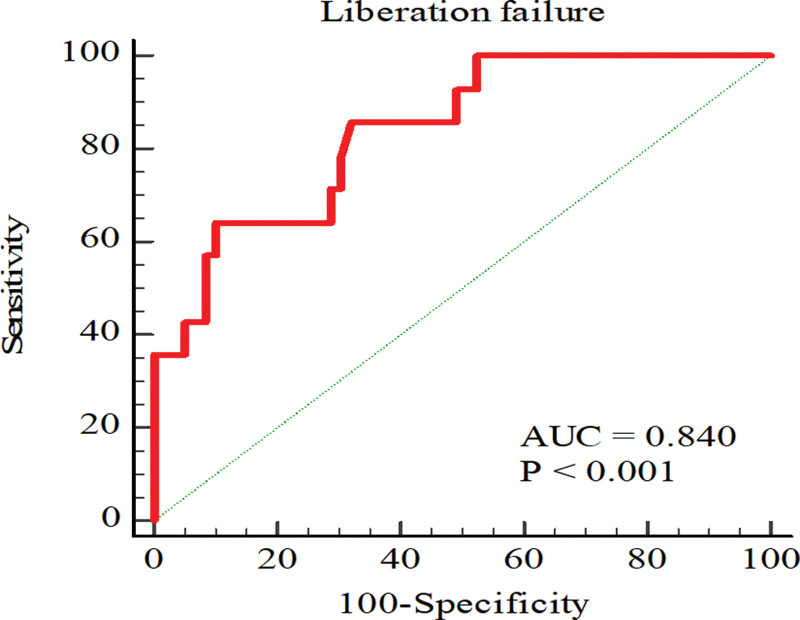
ROC curves to determine the significance of thoracic skeletal muscle index in predicting ventilator-weaning failure. ROC = receiver operating characteristic, TSMVI = total skeletal muscle volume index.

Table 2 shows the results of multivariate analysis for the predictors identified in the univariate logistic regression analysis. Sarcopenia was significantly associated with an increased risk of weaning failure (odds ratio [OR] 9.28, 95% CI 2.42–38.93, *P* = .001 in the univariate analysis and OR 12.72, 95% CI 2.87–70.48, *P* = .001 in the multivariate analysis). On the other hand, a higher SOFA score on the procedure day was an independent predictor of weaning failure (OR 1.35, 95% CI 1.04–1.79, *P* = .029 in the univariate analysis and OR 1.43, 95% CI 1.07–2.03, *P* = .024 in the multivariate analysis). Receiver operating characteristic curve analysis of TSMVI for weaning outcomes was used to predict weaning failure more accurately. The AUC of the TSMVI for predicting weaning failure was 0.840. The optimal cutoff value of 630 cm^3^/m^2^ had 64.3% sensitivity and 89.8% specificity (95% CI: 0.729–0.950, *P* < .001) (Fig. 4).

**Table 2:** Predictors associated with liberation failure in study patients.

Variables	Univariate		Multivariate	
	Odds ratio (95% CI)	*P* value	Odds ratio (95% CI)	*P* value
Sarcopenia	9.28 (2.42–38.93)	.001	12.72 (2.87–70.48)	**.001**
Sex (female)	1.19 (0.29–4.32)	.792		
Age, years	1.02 (0.98–1.07)	.429		
Body mass index, kg/m^2^	1.02 (0.05–7.18)	.987		
Cardiology cause	1.30 (0.06–9.86)	.824		
Pulmonary cause	2.07 (0.59–8.42)	.274		
SOFA at procedure day	1.35 (1.04–1.79)	.029	1.43 (1.07–2.03)	**.024**
LIS at procedure day	1.23 (0.53–2.80)	.620		

Bold type indicates statistical significance.

LIS = lung injury score, SOFA = sequential organ failure assessment.

## 4. Discussion

Identifying predictors of ventilator-weaning failure is of utmost importance because both premature weaning and PMV have been demonstrated to increase morbidity and mortality and incur significant financial and logistical burdens due to increased hospital costs and length of stay. The central finding of our study was that sarcopenia is associated with an increased incidence of weaning failure in patients with brain injury. To the best of our knowledge, this is the first study to demonstrate the usefulness of sarcopenia confirmed by the analysis of chest CT images for predicting failure of ventilator weaning in patients with brain injury.

Previous studies have found that pulmonary system-specific factors, such as tidal volume, minute ventilation, negative inspiratory force, and RSBI, were associated with weaning failure.^[13]^ However, these conventional weaning parameters have traditionally focused more on medical or surgical ICU patients and rarely include patients with brain injuries. Additionally, some studies have pointed out that traditional weaning parameters do not predict weaning failure in people with brain injuries.^[14]^ Indeed, it is not surprising that traditional weaning parameters do not predict weaning success, as intubation is often due to abnormal respiratory mechanics following brain injury rather than lung pathology.^[15]^ These is consistent with previous studies in which, despite a favorable weaning profile, a significant proportion of brain-injured patients are still considered difficult to liberate from MV. Consequently, it is essential to identify other predictors, in addition to existing weaning profiles, to avoid unnecessary or early ventilator weaning in patients with brain injuries.

In this analysis of a cohort of patients with brain injury, we found that several factors such as a higher SOFA score and sarcopenia were associated with weaning failure from MV. Even after adjusting for several risk factors associated with weaning failure, sarcopenia remained an independent predictor of weaning failure in this patient population. In addition, sarcopenia is associated with extended hospital stay in patients with brain injuries. Accordingly, our findings suggest that sarcopenia may predict adverse outcomes in patients with brain injury who are preparing for weaning from MV. In addition, our results are consistent with those of previous studies showing that sarcopenia is associated with adverse outcomes in critically ill patients. Therefore, given the high burden on these patients and the frequent poor outcome, it is essential to identify sarcopenia as a simple and useful predictor of ventilator-weaning failure.

The exact pathophysiological mechanism between sarcopenia and the increased risk of weaning failure in patients with brain injury remains unclear. Indeed, research on cerebrovascular dysfunction or brain lesions has been intensively conducted in patients with brain injuries such as stroke. However, systemic changes and dysregulation of peripheral organs in patients with brain injury are not well known. These peripheral organ changes include skeletal muscle loss and remodeling processes.^[16]^ As with other chronic diseases such as cancer or heart failure, brain injury also affects metabolic balance through free radical stress, significant elevations of proinflammatory cytokines, or activation of the neuroendocrine systems.^[17]^ Thus, an overall catabolic/anabolic imbalance can develop with increased catabolic and failed anabolic stimuli. This results in tissue wasting of fat and skeletal muscle, and clinical signs of sarcopenia in patients with brain injury. Previous studies have suggested that sarcopenia in critically ill patients impairs respiratory muscle function, potentially leading to MV weaning failure.^[18]^ In this study, our results demonstrated that sarcopenia has significant predictive power for weaning failure among patients with brain injury. In this regard, our findings suggest the importance of skeletal muscle mass during weaning from MV and the need for risk stratification before the weaning process begins among patients with brain injury. However, as mentioned above, the exact mechanism linking sarcopenia and poor weaning outcomes remains unclear. More extensive studies are required to elucidate this mechanism.

There is no gold standard method or reference value for defining sarcopenia in patients with brain injuries. In addition, since patients with brain injury are generally immobile or unconscious, accurate evaluation of sarcopenia is rarely possible and requires a more objective approach. The European Consensus Statement suggested that CT imaging could be used as a noninvasive assessment of sarcopenia in critically ill patients whose muscle mass, strength, and physical activity cannot be measured.^[4]^ Most studies that have used CT images to evaluate sarcopenia in critically ill patients have used cross-sectional areas of respiratory muscles using chest CT images. In contrast to previous studies, our study used the volume of the respiratory muscles at the thoracic vertebral level (T1–T12) using chest CT images. Indeed, evaluating sarcopenia using the respiratory muscle volume is better and more reliable than a single axial image because it can measure a broader respiratory muscle and reduce statistical error.^[19]^ In addition, since the analysis program automatically calculates the respiratory muscle volume, sarcopenia can be measured more simply and objectively. Other studies have shown that psoas muscle volume is better for identifying sarcopenia than cross-sectional area.^[20]^

Our study has some limitations. First, ideal cutoff values for defining sarcopenia in patients with brain injury have not yet been established. Following previous studies, we arbitrarily determined the cutoff value for sarcopenia in this study^.[21,22]^ Therefore, further studies are needed to assess the ideal cutoff for TMI values to define sarcopenia in patients with brain injury. Second, this study evaluated muscle volume only once using chest CT images upon admission to the ICU. Changes in muscle mass were not assessed during hospitalization. Therefore, it is impossible to determine whether muscle volume gain is conducive to weaning success, based on this study alone. Third, although we adjusted for factors contributing to weaning failure, the study was retrospective and may have hidden unknown predictors or confounding factors. Therefore, our findings should be cautiously interpreted. Fourth, the study was a single-institution observational retrospective analysis, and the number of enrolled patients was small. Further prospective multicenter studies with larger cohorts are required to validate these results.

Despite these limitations, our results provide valuable insights into the impact of thoracic skeletal muscle volume on weaning outcomes from MV in patients with brain injuries. This study is the first to demonstrate the usefulness of thoracic skeletal muscle volume as a risk assessment tool for ventilator weaning outcomes in patients with brain injuries. Despite comorbidities or higher SOFA scores, sarcopenia is associated with increased weaning failure rates. Therefore, the assessment of sarcopenia may contribute to benefit-risk stratification in patients with brain injury preparing for weaning from MV. Finally, thoracic skeletal muscle mass is modifiable and sarcopenia can be used to determine appropriate nutritional interventions, which may improve weaning outcomes in patients with brain injury.

## 5. Conclusions

Various factors related to success or failure are related to liberation from MV, and there are more complex and unexplained factors, especially in patients with brain injuries. Nevertheless, in our study, thoracic skeletal muscle volume is an independent predictor of MV weaning failure in patients with brain injury.

## Acknowledgments

We would like to thank Editage (www.editage.co.kr) for editing and reviewing this manuscript for the English language. This research was supported by Wonkwang University in 2022 and by a grant from the Korea Health Technology R&D Project through the Korea Health Industry Development Institute (KHIDI) funded by the Ministry of Health & Welfare, Republic of Korea (grant number: HI22C0787, HI18C1216).

## Author contributions

**Conceptualization:** Jimi Oh, Hyun Lim, Chang Won Jeong, Ui Ri An, Joo Un Park, Youe Ree Kim, Chul Park.

**Data curation:** Hyun Lim, Wu Seong Kang, Ui Ri An, Joo Un Park, Youngick Ahn, Chul Park.

**Formal analysis:** Jimi Oh, Hyun Lim, Min Su Kim, Jinseok Lee, Wu Seong Kang, Ui Ri An, Joo Un Park, Youngick Ahn, Chul Park.

**Funding acquisition:** Min Su Kim, Chul Park.

**Investigation:** Min Su Kim, Jinseok Lee, Wu Seong Kang, Chul Park.

**Methodology:** Jimi Oh, Chang Won Jeong, Min Su Kim, Chul Park.

**Project administration:** Chang Won Jeong, Min Su Kim, Jinseok Lee, Youe Ree Kim, Chul Park.

**Resources:** Chul Park.

**Software:** Chang Won Jeong, Youe Ree Kim, Chul Park.

**Supervision:** Jinseok Lee, Chul Park.

**Validation:** Chang Won Jeong, Youe Ree Kim, Chul Park.

**Visualization:** Youe Ree Kim, Chul Park.

**Writing – original draft:** Jimi Oh, Hyun Lim, Chang Won Jeong, Min Su Kim, Jinseok Lee, Wu Seong Kang, Ui Ri An, Joo Un Park, Youngick Ahn, Youe Ree Kim, Chul Park.

**Writing – review & editing:** Jimi Oh, Hyun Lim, Chang Won Jeong, Min Su Kim, Jinseok Lee, Wu Seong Kang, Ui Ri An, Joo Un Park, Youngick Ahn, Youe Ree Kim, Chul Park.
